# *Gladiolus hybridus* ABSCISIC ACID INSENSITIVE 5 (*Gh*ABI5) is an important transcription factor in ABA signaling that can enhance *Gladiolus* corm dormancy and *Arabidopsis* seed dormancy

**DOI:** 10.3389/fpls.2015.00960

**Published:** 2015-11-03

**Authors:** Jian Wu, Shanshan Seng, Juanjuan Sui, Eliana Vonapartis, Xian Luo, Benhe Gong, Chen Liu, Chenyu Wu, Chao Liu, Fengqin Zhang, Junna He, Mingfang Yi

**Affiliations:** ^1^Beijing Key Laboratory of Development and Quality Control of Ornamental Crops, Department of Ornamental Horticulture and Landscape Architecture, China Agricultural UniversityBeijing, China; ^2^Department of Biological Sciences, University of TorontoToronto, ON, Canada; ^3^Department of Cell and Systems Biology, University of TorontoToronto, ON, Canada; ^4^College of Horticulture, Sichuan Agricultural UniversityYa’an, China

**Keywords:** *Gladiolus hybridus*, corm, dormancy, *Gh*ABI5, ABA

## Abstract

The phytohormone abscisic acid (ABA) regulates plant development and is crucial for abiotic stress response. In this study, cold storage contributes to reducing endogenous ABA content, resulting in dormancy breaking of *Gladiolus*. The ABA inhibitor fluridone also promotes germination, suggesting that ABA is an important hormone that regulates corm dormancy. Here, we report the identification and functional characterization of the *Gladiolus* ABI5 homolog (*Gh*ABI5), which is a basic leucine zipper motif transcriptional factor (TF). *Gh*ABI5 is expressed in dormant vegetative organs (corm, cormel, and stolon) as well as in reproductive organs (stamen), and it is up-regulated by ABA or drought. Complementation analysis reveals that *Gh*ABI5 rescues the ABA insensitivity of *abi5-3* during seed germination and induces the expression of downstream ABA response genes in *Arabidopsis thaliana* (*EM1*, *EM6*, and *RD29B*). Down-regulation of *GhABI5* in dormant cormels via virus induced gene silence promotes sprouting and reduces the expression of downstream genes (*GhLEA* and *GhRD29B*). The results of this study reveal that *Gh*ABI5 regulates bud dormancy (vegetative organ) in *Gladiolus* in addition to its well-studied function in *Arabidopsis* seeds (reproductive organ).

## Introduction

Dormancy has been described as one of the least understood phenomena in plant biology and remains confusing in spite of much recent progress (Finkelstein et al., 2008). This reflects the fact that dormancy is a complex process with many contributing factors. Since plants often face harsh environmental conditions, they have evolved a number of traits that promote survival. For example, many plants undergo a period of temporary growth cessation known as dormancy (Finkelstein et al., 2008). It can occur in both reproductive organs (seed) and vegetative organs (bud or tuber), and is also crucial for organ development and architecture (Horvath et al., 2003;Graeber et al., 2012). In cereal crops, dormancy is an essential trait because it prevents pre-harvest sprouting. The lack of harvest dormancy (vivipary) in cultivars of maize or wheat has resulted in large economic losses (Suzuki et al., 2001;Yang et al., 2007). However, deep dormancy can also be problematic: in barley, it prevents grains from germinating in a rapid and uniform manner that is required in the process of malting (Millar et al., 2006). In the horticultural industry, deep dormancy is an issue, and some treatments are necessary to promote germination and prevent extra storage costs (Gubler et al., 2005).

In seeds, various hormones regulate dormancy and germination. Specifically, while gibberellic acid (GA) and brassinosteroids are reported to promote dormancy breaking, ABA is the primary positive regulator of seed dormancy (Finch-Savage and Leubner-Metzger, 2006; Rentzsch et al., 2012). Compared with seed dormancy, little is known about bud dormancy in perennial plants (Horvath et al., 2004;Cooke et al., 2012). For perennials, bud dormancy is commonly divided into three consecutive stages: para-dormancy (inhibition regulated by the apical bud), endo-dormancy (inhibition regulated by internal signals), and eco-dormancy (inhibition regulated by environmental factors) (Horvath et al., 2003; Anderson et al., 2010). However, *Gladiolus* corm dormancy differs slightly from common bud dormancy in perennials: after flowering in the summer, a new corm emerges and expands on the top of mother corm. In late autumn, the new corm and its cormels are in a state of endo-dormancy. Following 3–4 months of cold storage, the endo-dormant corm or cormel, transitions into the eco-dormant stage. During the latter stage, germination is affected by environmental factors such as light, temperature and water. In order to reduce the costs associated with cold storage, weaker dormancy is required for cultivators in the flower industry.

Although the molecular mechanism underlying the regulation of bud dormancy is not well-understood, advances that shed light on this physiological process have been made. A gene involved in the regulation of flowering, *FT* (*FLOWERING LOCUS T*), is reported to be involved in establishing dormant buds and in dormancy state transitions in poplar. Knocking down the expression of *FT* results in growth cessation and bud set (Bohlenius et al., 2006; Horvath, 2009). Moreover, transcriptome analysis of bud dormancy in leafy spurge, peach and pear led to the proposition that *DORMANCY ASSOCIATED MADS-BOX* (*DAM*) genes regulate bud dormancy-related processes (Horvath et al., 2008; Leida et al., 2012; Liu et al., 2012). Ectopic expression of *DAM* in *Arabidopsis* down-regulated *FT* expression (Horvath et al., 2010). In woody plants, *CBF* (*C-REPEAT/DRE BINDING FACTOR*), *DAM*, *RGL* (*RGA-LIKE*), and *EBB* (*EARLY BUD-BREAK*) genes have been shown to be associated with freezing tolerance, dormancy, and bud breaking (Wisniewski et al., 2015).

Abscisic acid is also a key factor that regulates dormancy. Short day length impacts the induction and maintenance of endo-dormancy by triggering ABA accumulation (Heide, 1986; Li et al., 2005). In autumn, short-term cold treatment also inhibits the growth of perennials by inducing endo-dormancy through the action of ABA and GA (Horvath et al., 2003). However, reducing ABA content in potato tubers results in endo-dormancy breaking, which may be due to the altered expression of *NCEDs* (*9-cis-epoxycarotenoid dioxygenases*) and *CYP707As* (*Cytochrome P450, Family 707, Subfamily A*) (Destefano-Beltran et al., 2006). Cold temperature has been reported to reduce endogenous ABA content, and contributes to dormancy breaking in lily, grape and pear (Xu et al., 2006; Liu et al., 2012; Zheng et al., 2015). ABA is commonly regarded as a stress hormone since it is involved in the response to drought and osmotic stress, abscission and the induction of dormancy (Finkelstein, 2013). The signal transduction pathway of ABA in the plant is clear: in the absence of ABA, PP2Cs (Type 2C Protein Phosphatases) are active and inhibit the activity of SnRK2 (SNF1-Related Protein Kinase 2), rendering it unable to regulate downstream factors, blocking ABA responses. In the presence of ABA, ABA receptors interact with PP2Cs and repress their phosphatase activity, thereby activating SnRK2. SnRK2 then phosphorylates bZIP TFs (e.g., ABI5) which activate the expression of ABA-response genes (Hubbard et al., 2010; Melcher et al., 2010).

ABI5 belongs to the bZIP superfamily group A owing to its structural features including conserved regions C1–C2, basic regions, and leucine zippers (Finkelstein and Lynch, 2000). *At*ABI5 plays a central role in regulating ABA-responsive genes in seeds by binding to ABA-responsive-elements (ABREs, ACGTGG/TC) in the promoter region of genes such as *AtEM6* (*EARLY METHIONINE-LABELED 6*), *AtRD29A* (*RESPONSIVE TO DESICCATION 29A*), *AtRD29B* and *AtRAB18* (*RAB GTPASE HOMOLOG B18*) (Carles et al., 2002; Miura et al., 2009). Phosphorylation by SnRK2 affects *At*ABI5 activation and stability, whereas ABI5 instability is promoted by SER/THR PROTEIN PHOSPHATASE 6 (Dai et al., 2013; Feng et al., 2014). Furthermore, *At*ABI5 is degraded by KEEP ON GOING (KEG; Stone et al., 2006). The transcriptional activation and protein stability of *At*ABI5 are also positively regulated by ABA (Lopez-Molina et al., 2001). It has been previously reported that rice *OsABI5-Like1* contributes to drought, salinity and osmotic stress responses mediated by ABA (Yang et al., 2011). However, research concerning the effects of ABI5 on bud dormancy is limited.

Through comparison of transcriptome data from dormant seed and bud in peach, some common features have been identified, including ABA response-related genes such as *ABI5*-like, *ABI3*-like, *ABI5 binding protein* (*AFP*)-like, and *LATE EMBRYOGENESIS ABUNDANT* (*LEA*)-like (Leida et al., 2012). AFP is believed to promote the degradation of *At*ABI5, and *AtABI3* acts directly upstream of *AtABI5* to positively regulate *AtABI5* expression (Finkelstein and Lynch, 2000; Lopez-Molina et al., 2002, 2003; Dai et al., 2013). Although the effects of ABI5 on seed dormancy have been well-characterized, no direct genetic evidence yet exists to support the idea that ABI5 also functions in bud dormancy (Piskurewicz et al., 2008; Rodriguez et al., 2009; Nonogaki, 2014).

The purpose of the present study was to characterize the expression of *GhABI5* in vegetative dormant organs and to elucidate how it regulates corm dormancy.

## Materials and Methods

### Plant Material, Treatments, and Quantification of Endogenous Hormones

*Gladiolus hybridus* (‘Rose supreme’) was planted in the Science Research Garden at China Agricultural University on April 30th, 2012 and corms were harvested on October 30th. The corms were washed and dried at 25°C for 6 weeks, after which the cormels were separated from the corms. The cormels were randomly divided into two batches: one was stored in nylon mesh bags at 22 ± 1°C and the other was stored at low temperature (4 ± 1°C) in the refrigeration house; relative humidity was kept at 60–70%. Cormels for qRT-PCR and phytohormone quantification were sampled at 14-days intervals (0–12 weeks) during drought (0–6 weeks) and storage (6–12 weeks). GA and ABA content were measured using the indirect enzyme linking immunosorbent assay (ELISA) method (Yang et al., 2001), following the manufacturer’s instructions (Chemical Control Laboratory of Agriculture and Biotechnology College, China Agricultural University). Cormel tissue (0.2 g fresh weight) was immediately frozen in liquid nitrogen and stored at -80°C prior to hormone extraction. The tissue was homogenized in extraction solution (80% methanol (v/v) including 1 mM butylated hydroxytoluene as an antioxidant) and centrifuged at 10,000 *g* for 20 min at 4°C. The supernatant was then extracted at 4°C for 8 h.

Dormant cormels used for hormone treatments measured 0.8–1.0 cm in diameter and had been stored at 4°C for 2 weeks. These cormels were treated with ABA (25 μM), fluridone (25 μM) or distilled water before being placed in a light homoeothermic incubator at 25°C with 16/8 h light/dark. The tunic was removed from cormels used for drought treatment, which were left to break dormancy at room temperature for 1, 3, 6 days. The experiment were repeated with three biological replicates (*n* = 48).

Dormant cormels stored at 4 or 22°C for different lengths of time were sterilized and plated on 0.6% agar. They were then placed in a light incubator with 16/8 h light/dark for sprouting. The sprouting percentage was counted on the 21st day after planting. Fifty cormels per sample were used for each sprouting test. Error bars represent the SE of three biological repeats.

*Arabidopsis* seeds were sterilized with 1% (v/v) NaClO for 30 min, washed five times with sterile distilled water, and plated on MS medium. Following stratification for 3 days at 4°C in the dark, the seeds were transferred into a light homoeothermic incubator (22°C) with 16 h/8 h light/dark. Seven day-old seedlings were transferred to forest soil and vermiculite (1:1, v/v) in a greenhouse at 22°C with 16 h light/8 h dark under 50 μmol m^-2^ s^-1^ light. *Nicotiana benthamiana* seeds were planted in sterilized forest soil and vermiculite (1:1, v/v) and cultured under the same conditions as the *Arabidopsis* seedlings.

### Cloning and Sequence Analysis of *GhABI5*

Total RNA from dormant *Gladiolus* cormels was extracted using the Tiangen RNA extraction reagent kit (Tiangen, China). Subsequently, cDNA was synthesized by M-MLV reverse transcriptase (TaKaRa, Japan) with a 3′ adaptor (5′-CCAGTGAGCAGAGTGACGAGGACTCGAGCTCAAGC(T)_17_-3′).

Degenerate primers, *Gh*ABI5-DP-for (5′-GGBTCCATGAACATGGACGAG-3′) and *Gh*ABI5-DP-rev (5′-KCCAVACCTCVTCVACVGTCTT-3′), were designed for *GhABI5* cloning based on conserved regions of homologous ABI5 genes from *Arabidopsis thaliana*, *Triticum aestivum*, *Hordeum vulgare*, and *Zea mays* (Supplementary Table S2). The amplified product was used to clone the full-length *GhABI5* sequences by the Rapid Amplification of cDNA Ends (RACE) method and nested PCR according to the manufacturer’s protocol (Clontech, Mountain View, CA, USA). All PCR products were subcloned into the pMD18-T vector (TaKaRa, Japan) and transformed into *E. coli* DH5α cells (Trans, China) and sequenced. Primer sequences are listed in Supplementary Table S1.

Protein characteristics were analyzed with the ExPASy Proteomics Tools.^1^ ClustalX1.8 and BioEdit7.0 were used to perform multiple amino acid alignments, and phylogenetic trees were constructed by the neighbor-joining method using the MEGA5.0 software (Tamura et al., 2011). Accession numbers for the public sequences used in the phylogenetic analysis are listed in Supplementary Table S2.

### *GhABI5* Promoter Isolation and Petal Transient Expression

The upstream regulatory sequence of *GhABI5* was isolated using high-efficiency thermal asymmetric interlaced PCR (hiTAIL-PCR; Liu and Chen, 2007). The position of the translation start site was designated as “+1”. The cis-regulatory elements were analyzed and annotated using the software programs from the Plant *Cis*-acting Regulatory DNA Elements database^2^ (PLACE). The 1688 bp upstream regulatory sequence of *GhABI5* was inserted into the pCAMBIA1391 binary vector with *Hin*dIII and *Bam*HI restriction sites. This *GhABI5:GUS* plasmid was then introduced into *Agrobacterium tumefaciens* strain GV3101 and injected into *G. hybridus* petals (Batoko et al., 2000). The petals were cultured with water or 50 μM ABA, and covered with preservative film. Petal disks were GUS-stained after 2 days, following a previously established protocol for GUS histochemical staining (Jefferson, 1987).

### Quantitative RT-PCR Analysis

About 400 ng cDNA was used as the template for qRT-PCR using the Applied Biosystems StepOnePlus^TM^ real-time PCR system with the KAPA^TM^ SYBR^®^ FAST qPCR kit (Kapa Biosystems, Woburn, MA, USA). The *Gladiolus* actin gene (GenBank accession no. JF831193) and *Arabidopsis Actin2* (GenBank accession no. NM-112764) were used as controls in *Gladiolus* and *Arabidopsis*, respectively. The PCR procedure was performed according to the manufacturer’s instructions. Each qRT-PCR assay was run in three biological replicates. Primer sequences are listed in Supplementary Table S1.

### Subcellular Localization of *GhABI5*

The open reading frame (ORF) of *GhABI5* was cloned into pCAMBIA1300-GFP with *Xba*I and *Kpn*I restriction sites. Both the fusion construct (*GhABI5-GFP*) and the control (*GFP*) were transformed into onion epidermal cells by particle bombardment. After incubation for 20 h at 25°C in the dark, the cells were observed under bright field and fluorescence using confocal microscopy (Nikon Inc., Melville, NY, USA). The vectors were also transformed into *Agrobacterium tumefaciens* GV3101 and used to agroinfiltrate *N. benthamiana* leaves for transient expression, as described previously (Batoko et al., 2000). After 3 days, the leaves were observed in the same manner as the onion cells.

### Determination of Dormancy in *Arabidopsis* Overexpressing *GhABI5*

*Arabidopsis* plants (*abi5-3* and Col-0) were transformed using the floral dip method (Clough and Bent, 1998) with *Agrobacterium tumefaciens* GV3101 harboring *Gh*ABI5 fused with GFP at its N-terminus (GFP-*Gh*ABI5/pCAMBIA1300).

Infected seeds were collected and sterilized with 1% (v/v) NaClO for 30 min, washed with distilled water and plated on MS medium containing 50 μg l^-1^ hygromycin B (Roche, Germany) before stratification for 3 days at 4°C. The plates were then transferred into the growth chamber to select transformed seedlings. T3-generation homozygous seeds were used for all experiments.

To ensure all freshly harvested seeds were of similar maturity, we carefully selected early siliques of the same yellow color. Seeds were sown directly on water-saturated filter paper, with or without ABA, to allow for germination under 16/8 h light/dark conditions at 22°C without stratification. Seeds were considered germinated once green cotyledons emerged, and germinated seeds were scored at the indicated times. Statistical analysis was performed with three biological replicates.

### Silencing of *GhABI5* in Dormant Cormels by VIGS

Silencing of *GhABI5* by virus-induced gene silencing (VIGS) was performed as described previously (Zhong et al., 2014), with some modifications. The pTRV1, pTRV2, and pTRV2-*GhABI5* (specific fragment of about 344 bp) vectors were transformed individually into *Agrobacterium tumefaciens* GV3101. The transformed *Agrobacterium* lines were cultured overnight in Luria-Bertani (LB) medium supplemented with 50 μg ml^-1^ kanamycin and 100 μg ml^-1^ rifampicin. The next day, the cells were collected and re-suspended in infiltration buffer (10 mM MgCl_2_, 200 mM acetosyringone, 10 mM MES, pH 5.6) to a final OD_600_ of about 2.0. Equal volumes of TRV1 and TRV2 (used as the control), as well as TRV1 and TRV2-*GhABI5* were mixed together and placed at room temperature for 3 h in the dark prior to vacuum infiltration.

Dormant corms (0.8–1.0 cm in diameter, stored for 2 months at 4°C) were first poked with pins on their top and bottom. Subsequently, they were subjected to vacuum infiltration under 0.7 MPa for 30 min. The cormels were then planted in pots and kept at 20°C for 7 days in darkness before cultivation in a growth chamber at 20°C with 16/8 h light/dark. The plants were measured with a vernier caliper after 10 days, and images were taken with a digital camera (Canon D500). The experiments were repeated three times using at least 96 cormels in each repetition. Duncan’s multiple range test (^∗^*P* < 0.05, ^∗∗^*P* < 0.01) was used for statistical analysis.

## Results

### Endogenous ABA and GA Content during Postharvest Storage of *Gladiolus*

**FIGURE 1:**
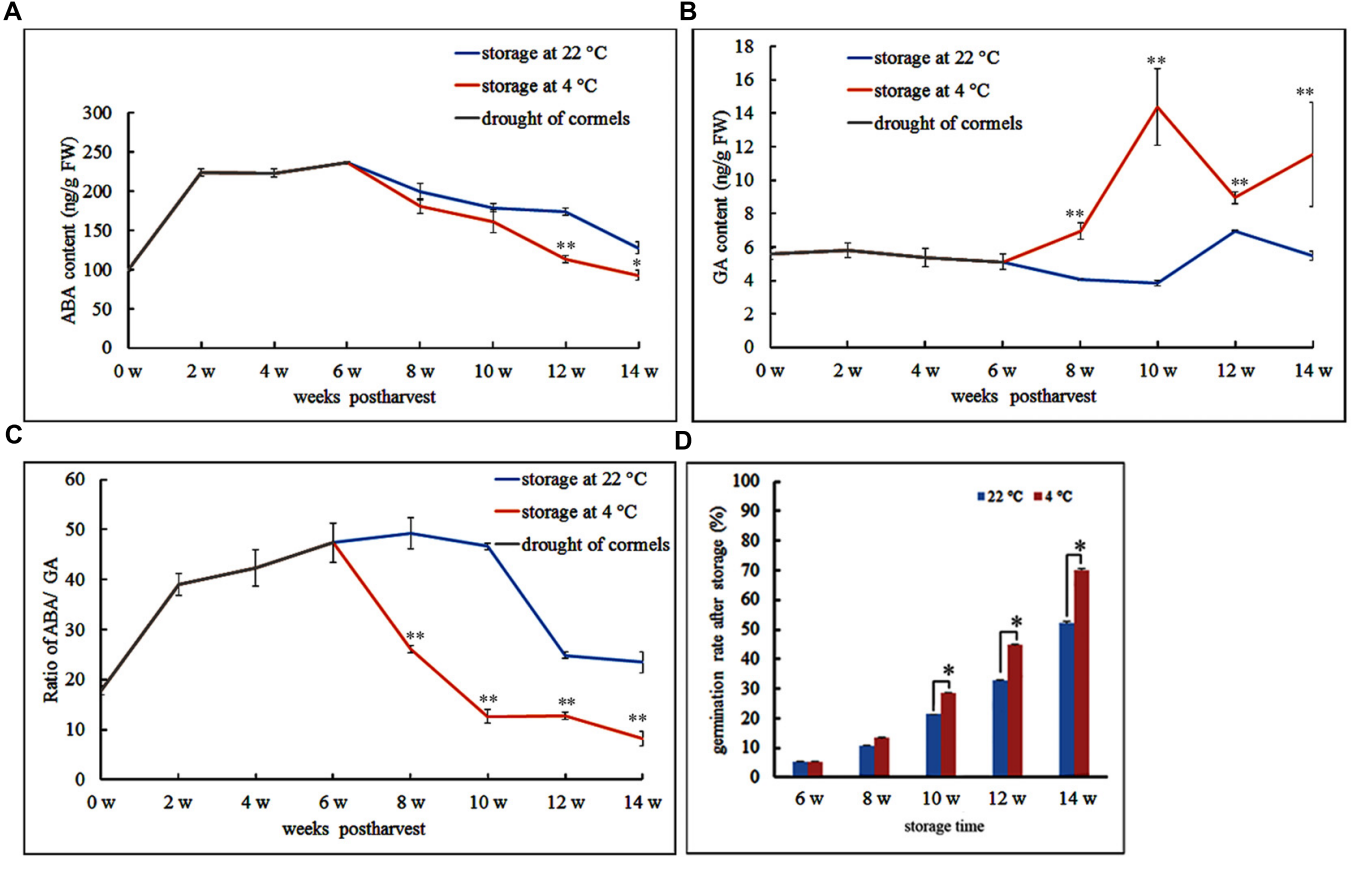
**Endogenous abscisic acid (ABA) and gibberellic acid (GA) levels in *Gladiolus* cormels under different temperature storage**. Postharvest storage is divided into two stages: drought of cormels (6 weeks) and then storage at 4°C or 22°C. **(A)** ABA changes in drought of cormels (0–6 w) and different storage temperature (6–14 w). **(B)** GA changes in drought of cormels (0–6 w) and different storage temperature (6–14 w). **(C)** Ratio of ABA/GA changes in drought of cormels (0–6 w) and different storage temperature (6–14 w). **(D)** The effect of temperature and duration on the sprouting of cormels. Error bars represent the SE of three replicates. (^∗^*P* < 0.05; ^∗∗^*P* < 0.01).

Abscisic acid is believed to be the main phytohormone that controls seed dormancy. Therefore, we wished to track fluctuations in ABA levels during *Gladiolus* dormancy breaking. We quantified ABA and GA levels during different time points throughout the desiccation and storage periods of freshly harvested corms. For the first 6 weeks, the freshly harvested cormels were dried in a well-ventilated and dry environment; during this period, endogenous ABA dramatically increased and reached its peak, while GA content somewhat decreased (**Figures 1A,B**) and the ratio of ABA to GA sustainably increased (**Figure 1C**). Meanwhile, we observed that after 6 weeks, the cormels were covered by a tunic that prevents further water loss and may protect internal organs.

To compare the effects of different storage temperatures on dormancy breaking, we measured ABA and GA content under storage at 4 and 22°C. Following the first 6 weeks, one batch of cormels was stored at 22°C. During this storage stage, ABA content showed a steady decrease, whereas GA levels were lowest at 10 weeks and peaked at 12 weeks post-harvest (**Figures 1A,B**). The ABA/GA ratio was highest at 10 weeks and then decreased dramatically (**Figure 1C**). However, low temperature helped reduce ABA content and increased GA content in the cormels stored at 4°C (**Figures 1A,B**). Thus, the ratio of ABA to GA is higher at 22°C than at 4°C (**Figure 1C**). We also tracked cormel sprouting under different storage temperatures. Our data shows that the cormels sprouted with greater frequency when stored for a longer period of time. In this case, storage at 4°C can accelerate germination compared with 22°C (**Figure 1D**).

### Effect of ABA and Fluridone on *Gladiolus* Corm Dormancy

**FIGURE 2:**
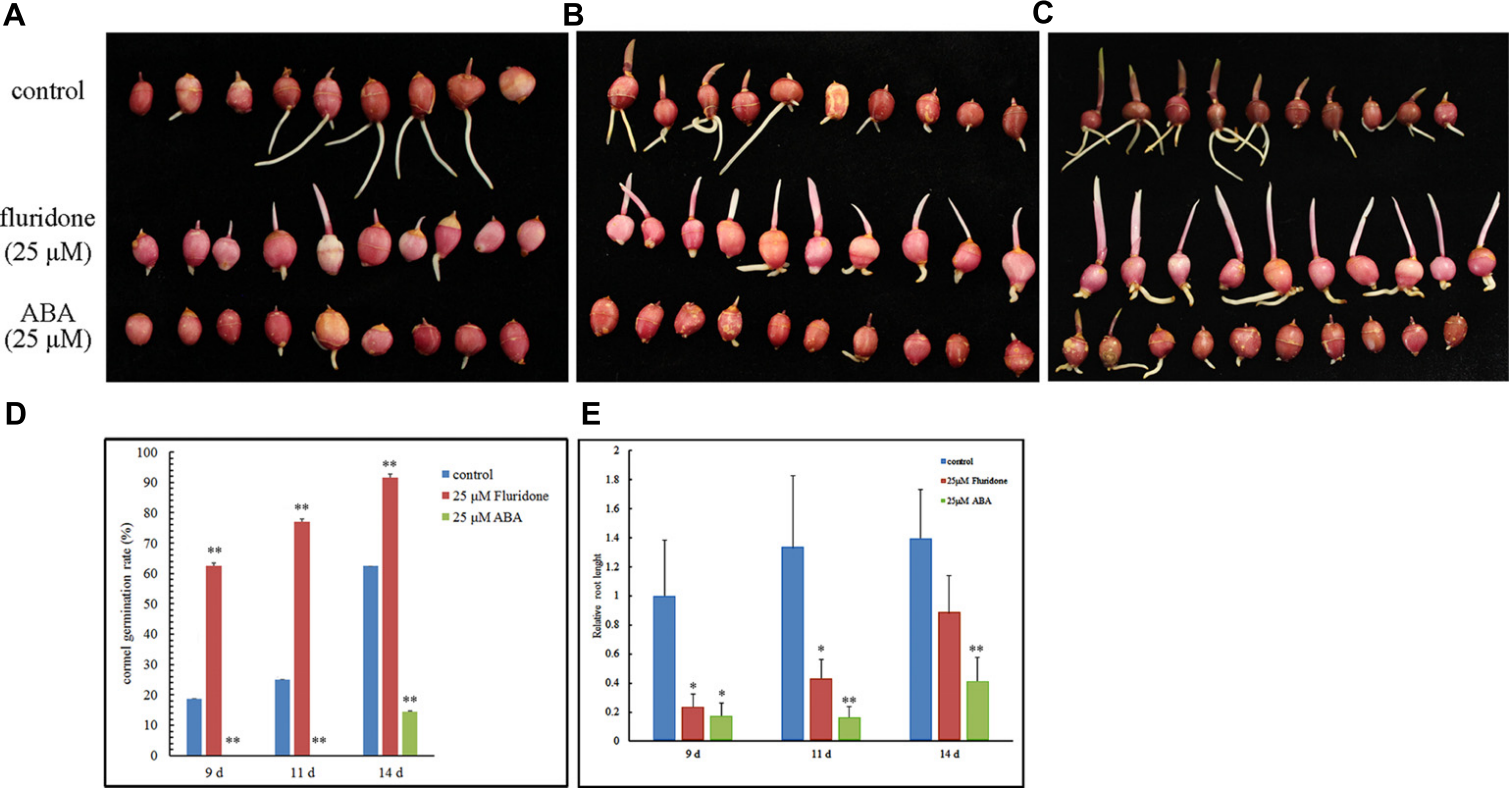
**Effects of exogenous ABA and fluridone on releasing *Gladiolus* corm dormancy. (A–C)** Cormels were submerged in water (control treatment), 25 μM fluridone or 25 μM ABA. Photos were taken at 9, 11, and 14 days post-treatment, respectively. **(D)** Germination (emergence of 1 cm buds) of cormels placed in water, 25 μM fluridone or 25 μM ABA. **(E)** The relative root length of cormels. Forty-eight cormels were used in each experiment. Error bars represent the SE of three biological replicates. (^∗^*P* < 0.05; ^∗∗^*P* < 0.01).

To further examine the role of ABA in corm dormancy (vegetative organ), corms were treated with exogenous ABA and fluridone. The results show that 62.5% of cormels treated with fluridone for 9 days germinated, compared with 18.75% of the control cormels (**Figures 2A,D**). Moreover, none of the ABA-treated cormels germinated. After 11 days, about 75% of fluridone-treated cormels germinated, while only 25% of the control and none of the cormels treated with ABA germinated (**Figures 2B,D**). After 13 days of fluridone treatment, almost all cormels had germinated. However, the germination rate for the control and ABA-treated cormels was roughly 62.5 and 15%, respectively (**Figures 2C,D**). These results show that ABA significantly inhibits the emergence and elongation of *Gladiolus* buds and roots (**Figure 2E**), thereby forcing *Gladiolus* to maintain dormancy. Fluridone effectively promotes sprouting but has an inhibitory effect on root elongation (**Figure 2E**). Taken together, the results illustrate that exogenous ABA is sufficient to repress germination and maintain *Gladiolus* corm dormancy.

### Isolation and Sequence Analysis of an ABA-responsive bZIP Transcription Factor

In order to better understand the role of ABA signaling in *G. hybridus* corm dormancy, we cloned a transcription factor belonging to the bZIP family. A conserved cDNA fragment was amplified using degenerate primers, and the full-length sequence was then isolated by the rapid amplification of cDNA ends (RACEs) method. The ORF of this bZIP transcription factor is 1221 bp in length, encoding a protein of 406 amino acid residues (AARs), with a calculated molecular mass of 44.53 KDa and an isoelectric point (p*I*) of 5.65.

**FIGURE 3:**
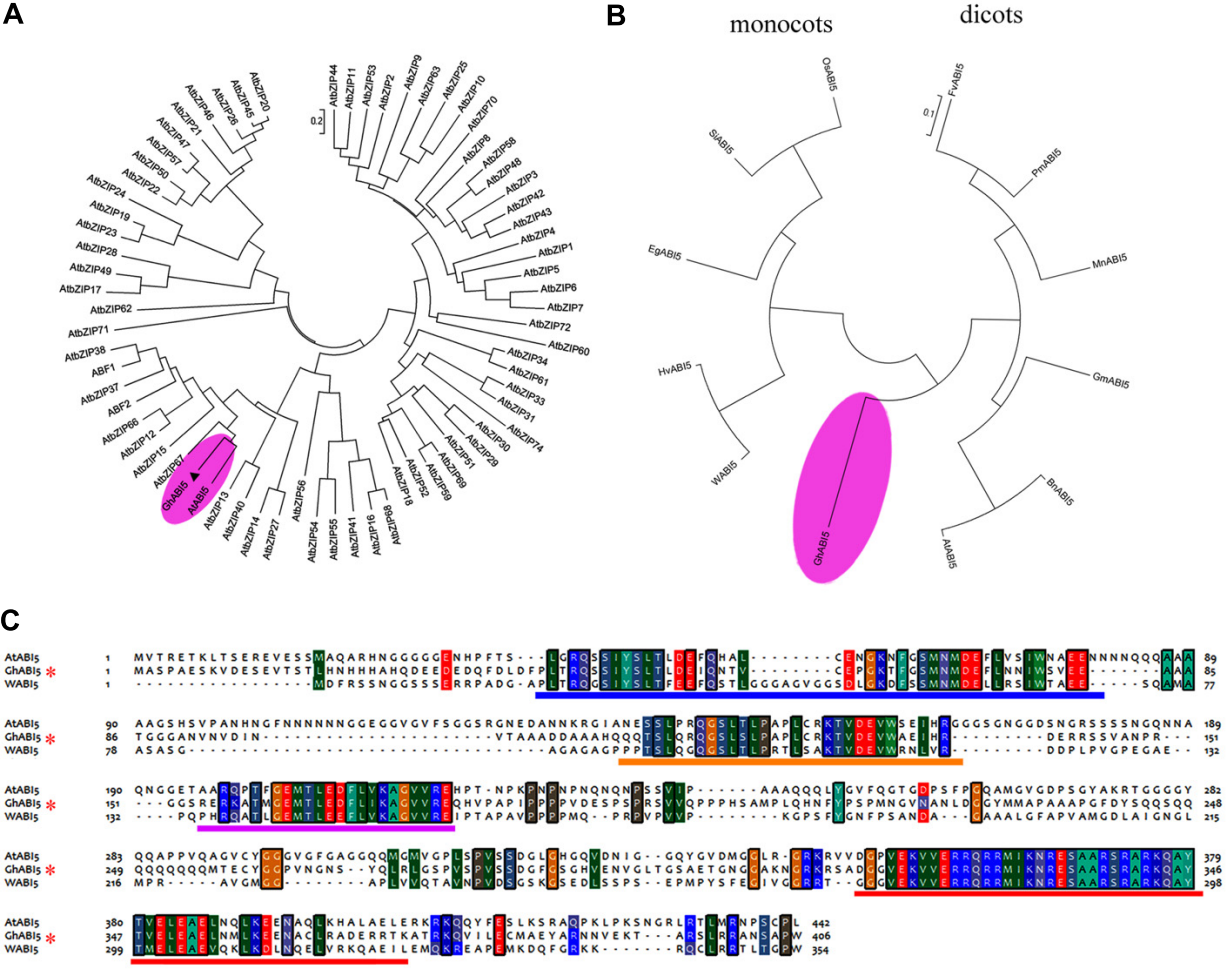
**Phylogenetic analysis of *Gh*ABI5. (A)** Phylogenetic relationships among *Gh*ABI5 and *Arabidopsis* bZIP proteins. **(B)** Phylogenetic analysis of *Gh*ABI5 and ABI5 homologous genes in other species. **(C)** Sequence alignment of *Gh*ABI5 with its homologs from *Arabidopsis thaliana* (*At*ABI5) and *Triticum aestivum* (*W*ABI5). The conserved C1, C2,C3, and bZIP domains are indicated by a blue, orange, purple, and red line, respectively. Information on the homologous ABI5 proteins is listed in Supplementary Table S2.

To investigate the characteristics of this gene, we performed a phylogenetic analysis using MEGA 5. The results (**Figure 3A**) suggest that our gene clusters closely with *AtABI5*. In addition, it shares more similarity with *AtABI5* than with the other 72 members of the *Arabidopsis* bZIP family^3^; therefore, we named the gene *GhABI5* (GenBank accession: KC344270). Furthermore, *GhABI5* forms its own clade and clusters more closely with the dicot genes than the monocot genes. (**Figure 3B**). *Gh*ABI5 is similar to *Gm*ABI5 from *Glycine max* (45% identity), *Hv*ABI5 from *Hordeum vulgare* (42% identity), WABI5 from *T. aestivum* (42% identity), *Fv*ABI5 from *Fragaria vesca* (41% identity), and *At*ABI5 from *Arabidopsis thaliana* (40% identity). Sequence alignment analysis (**Figure 3C**) shows that *Gh*ABI5 contains all distinguishing features of ABI5 proteins, including the conserved domains C1 (AARs 38 to 83), C2 (AARs 111 to 138), C3 (AARs 157 to 178), and basic domain (AARs 324 to 344) which is necessary for DNA binding (Finkelstein and Lynch, 2000; Zhou et al., 2013).

### Expression of *GhABI5* in Cormels during *Gladiolus* Dormancy Release

**FIGURE 4:**
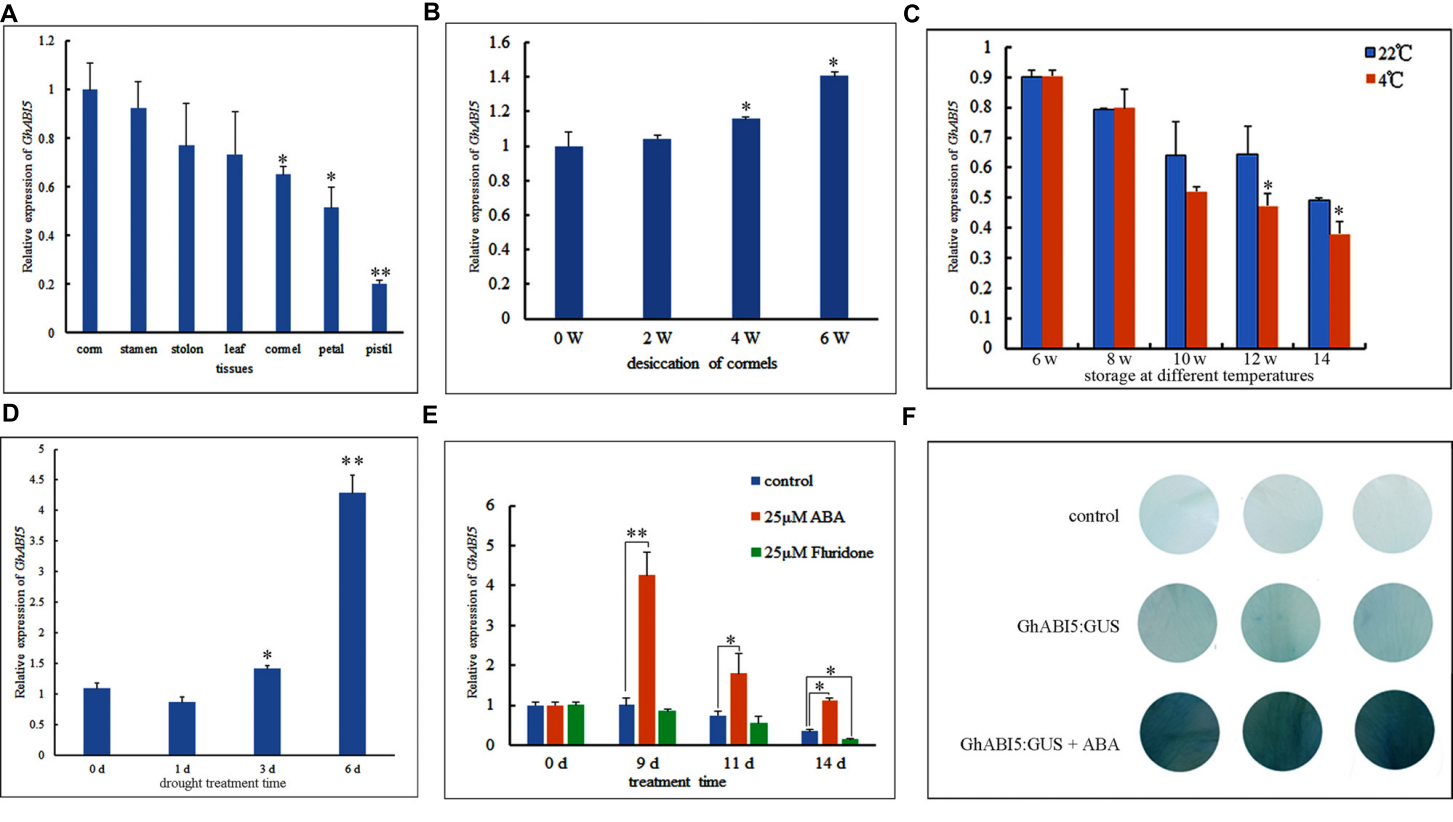
**Quantitative real-time PCR analysis of *GhABI5* expression levels and *GhABI5* promoter analysis. (A)** *GhABI5* expression levels in various organs. **(B)** *GhABI5* expression during storage at 22°C. **(C)** The effect of temperature and duration on the expression of *GhABI5*. **(D)** The expression of *GhABI5* under drought treatment (0, 1, 3, 6-days); **(E)** The expression of *GhABI5* in cormels treated with ABA and fluridone (0, 9, 11, 14-days). **(F)** Transient expression of *GhABI5:GUS* in *Gladiolus* petal disks with 50 μM ABA. Error bars represent the SE of three biological replicates. (^∗^*P* < 0.05; ^∗∗^*P* < 0.01).

Initially, *AtABI5* was thought to be expressed specifically in the seed; however, recent research has shown that *OsABI5* is also expressed in flowers (Zou et al., 2008) and in response to stresses (Lopez-Molina et al., 2003). Here, we analyzed the tissue-specific expression of *GhABI5* by real-time PCR. We found high expression levels in the corm, suggesting that *Gh*ABI5 may play an important role in this vegetative organ (**Figure 4A**). Then, we tracked *GhABI5* expression during the course of desiccation (0 to 6 weeks) and storage (6 to 14 weeks). The data (**Figure 4B**) showed increasing *GhABI5* expression during desiccation, reaching a peak at the end of this water loss period. *GhABI5* expression then gradually decreased during storage (**Figure 4C**). These results were consistent with the measured levels of ABA, suggesting that *GhABI5* expression is likely regulated by endogenous ABA.

In our study, we have shown that cold storage (4°C) helps *Gladiolus* break dormancy (**Figure 1D**). Therefore, we investigated the effect of temperature on *GhABI5* expression. Cold storage significantly repressed *GhABI5* expression at the two latest time points, compared with a higher temperature (22°C) (**Figure 4C**). This finding suggests that cold treatment can regulate *GhABI5* expression for a relatively short period of time. Furthermore, we found that *GhABI5* was highly responsive to drought, and its expression levels greatly increased 6 days following treatment (**Figure 4D**). Next, we analyzed the effect of ABA and the ABA inhibitor fluridone on the expression of *GhABI5*. Our data (**Figure 4E**) revealed that *GhABI5* was sharply up-regulated by 25 μM ABA treatment after 9 days, but 25 μM fluridone down-regulated *GhABI5* at 14 days. Fluridone may affect the metabolism of other hormones and indirectly influence *GhABI5* expression. Alternatively, there may be feedback regulation between *GhABI5* and ABA metabolism.

### Putative *cis*-acting Elements Identified in the *GhABI5* Promoter Region

Around 1.7 kb of the 5′ regulatory region upstream of the *GhABI5* transcription start site was isolated by hiTAIL-PCR. The acquired sequence was then scanned using the Plant *cis*-acting regulatory DNA elements (PLACE) database to obtain putative *cis*-regulatory elements potentially involved in the regulation of *GhABI5* expression. The data revealed a large number of elements that are putative target motifs for transcription factors reported to respond to hormones and hormone-related stresses (Supplementary Table S3 and Figure S1). Among these elements, ABA-related motifs are the most abundant: E-box/ABRE, CTR/DRE, low-temperature-responsive elements (LTREs) and RY/G box (an ABI3-binding motif). Moreover, GA and auxin response elements were also present in the promoter region (Supplementary Table S3). To test whether the *GhABI5* promoter is responsive to ABA, a transient expression in *Gladiolus* petal disks was performed through agroinfiltration (Batoko et al., 2000). The results show that ABA induces *GhABI5:GUS* expression (**Figure 4F**), confirming the result that *GhABI5* is up-regulated by ABA (**Figure 4E**).

### Subcellular Localization of *Gh*ABI5

**FIGURE 5:**
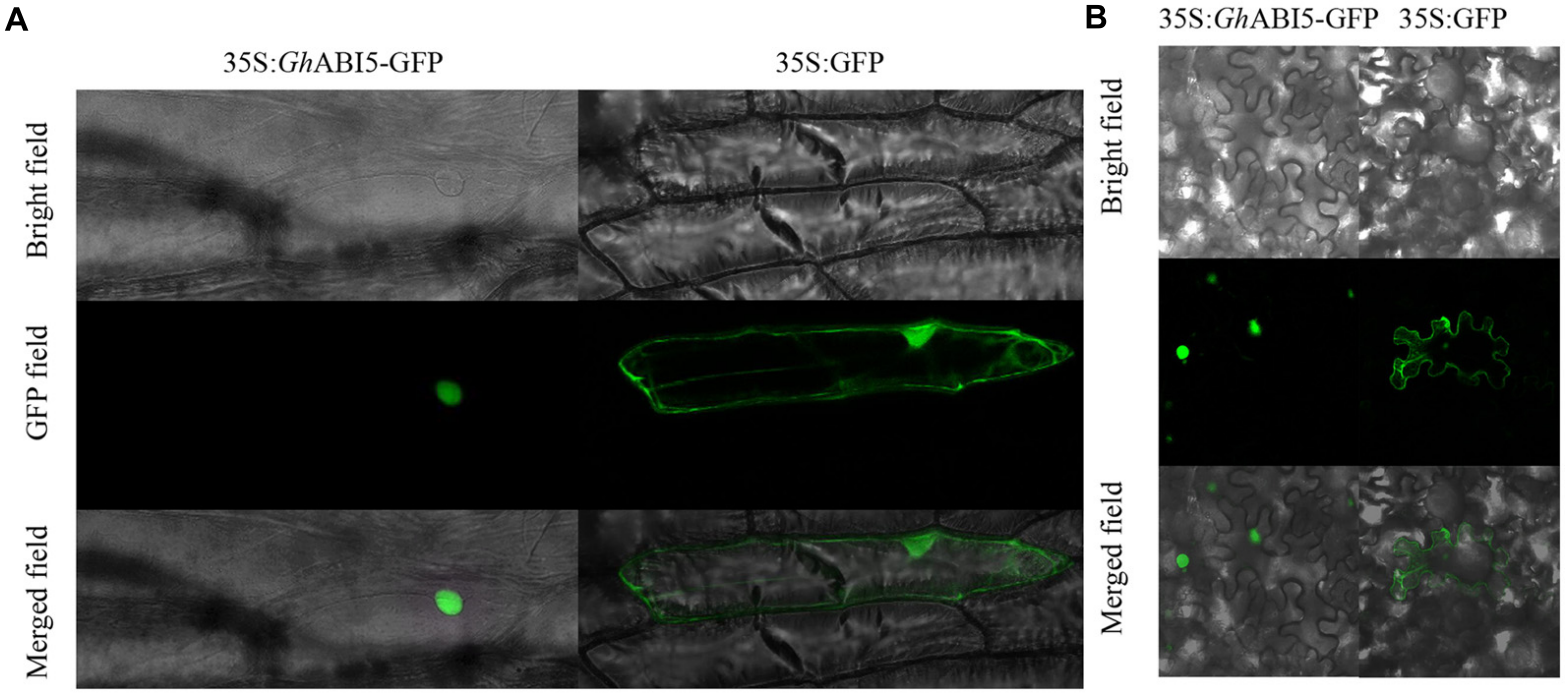
**The nuclear localization of GhABI5. (A)** The subcellular localization of *Gh*ABI5 in onion epidermal cells. **(B)** The subcellular localization of *Gh*ABI5 in *Nicotiana benthamiana* epidermal cells. *35S:GFP* was used as the control.

To learn more about *Gh*ABI5, we investigated its subcellular localization using both onion and *N. benthamiana* epidermal cells. For this purpose, we fused the ORF of *Gh*ABI5 to GFP. The recombined plasmid was transformed into onion epidermal cells by particle bombardment. In the epidermal cells expressing GFP-*Gh*ABI5, the GFP signal was present in the nucleus, while the 35S:GFP signal could be seen throughout the cells (**Figure 5A**). The same results were obtained in the *N. benthamiana* transient expression system (**Figure 5B**), which had been transformed using *Agrobacterium*. These results confirm that *Gh*ABI5 is a nuclear protein.

### Enhancement of Dormancy in *Arabidopsis* Overexpressing *GhABI5*

**FIGURE 6:**
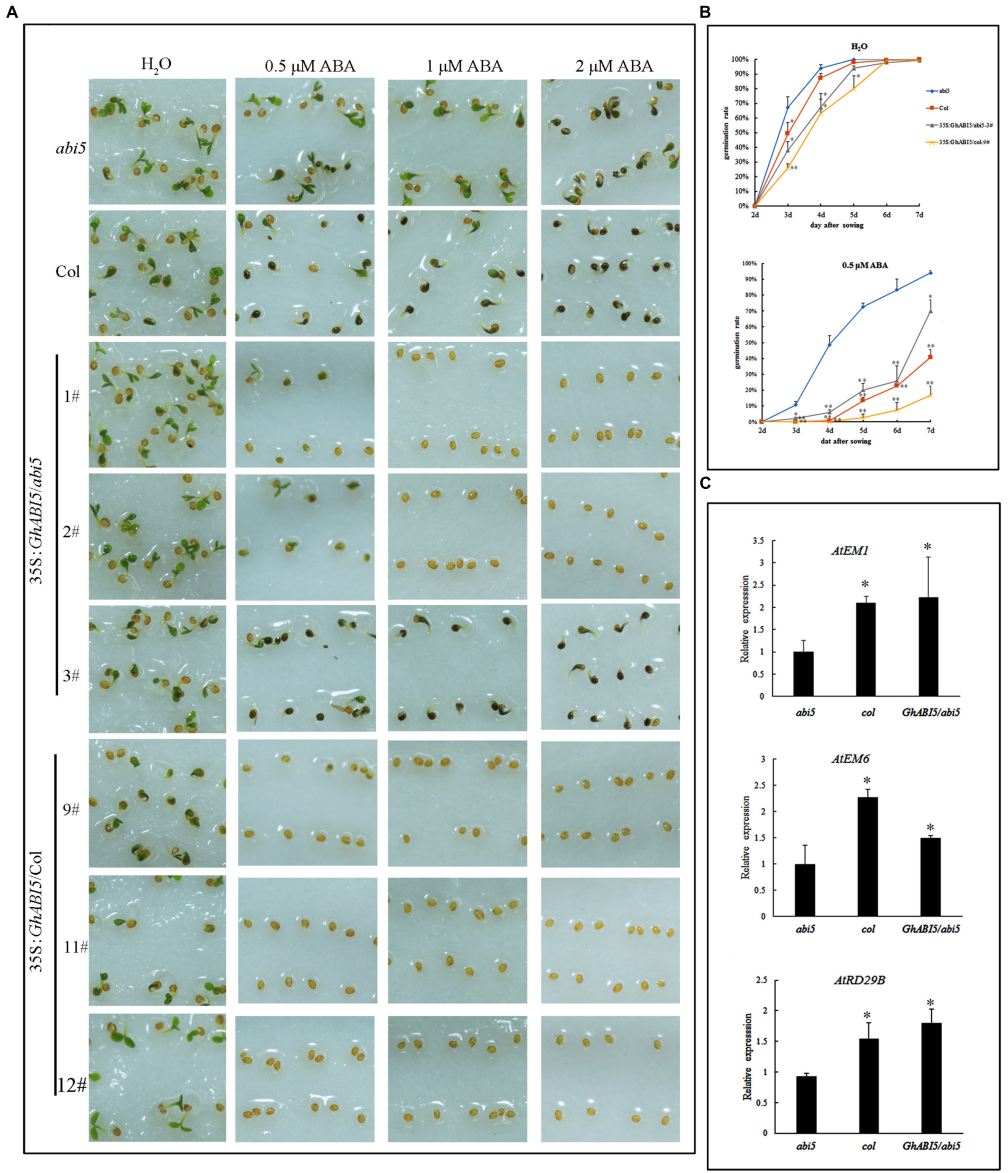
***GhABI5* responds to ABA, inhibiting seed germination and inducing downstream gene expression. (A)** Transgenic plants overexpressing *GhABI5* are hypersensitive to ABA. Seeds of *abi5-3*(*abi5*), Col, *GhABI5* complemented lines (1, 2, 3#) and overexpression lines (9, 11, 12#) were sown on filter paper with different concentrations of ABA without stratification, and photographed after 2 days. **(B)** Germination rate of transgenic seeds (35S:*GhABI5*/*abi5*-1# and 35S:*GhABI5*/Col-9#), *abi5* and Col. Without ABA, the germination rate is similar among the lines, compared with the different rates resulting from 0.5 μM ABA treatment. A seed with open green cotyledons is considered germinated. **(C)** *Gh*ABI5 positively regulates the expression levels of ABA-responsive genes. The freshly harvested seeds, including complemented transgenic seeds (1, 2, 3#), *abi5* and Col, were used for qRT- PCR. Bars represent the SE of three biological replicates. (^∗^*P* < 0.05; ^∗∗^*P* < 0.01).

The role of *GhABI5* in dormancy was also examined by the ectopic expression of 35S:*GhABI5* in *abi5-3* and wild type (Col) (Supplementary Figure S2). Without ABA treatment, the transgenic seeds exhibited a somewhat lower germination rate compared with Col (**Figures 6A,B**). Furthermore, when grown on media containing 0.5 μM ABA for 6 days, 57% of *GhABI5* complemented *abi5-3* seeds were sensitive to ABA while *abi5*-*3* was still insensitive to ABA (**Figure 6B**). In addition, on 1 μM ABA, seeds of 35S:*GhABI5*/Col and two complemented lines did not germinate, whereas seeds of the weakly complemented line showed similar ABA sensitivity to that of Col. Comparable results were observed with the 2 μM ABA treatment (**Figure 6A**). These results indicate that with exogenous ABA treatment, *GhABI5* contributes to seed dormancy, thereby enhancing the dormancy of *Arabidopsis* seeds. *GhABI5* also rescues the dormancy in complemented lines. Seeds overexpressing *GhABI5* in the Col-0 background exhibit deeper dormancy than the *abi5-3* lines complemented with *GhABI5*.

To further confirm whether *GhABI5* functions as an ABA response transcription factor and has conserved functions similar to *AtABI5*, we analyzed the genes downstream of *AtABI5* in freshly harvested, complemented mature dormant seeds. The results of the qRT-PCR analysis show that transcript levels of *EM1*, *EM6*, and *RD29B* were up-regulated to various degrees in *GhABI5* complemented lines (**Figure 6C**). EM1 is an ABA-inducible protein that accumulates during seed maturation; in the complemented lines, its expression recovered to a level similar to Col, and was 2.3-fold greater than in the *abi5-3* mutant. The transcript level of *EM6* increased 1.5-fold but could not recover to the wild type level. The expression of *RD29B* in complemented lines increased 1.9-fold compared with *abi5-3*. Overall, the transcript levels of genes downstream of *AtABI5* were up-regulated in the *abi5-3* lines complemented with *GhABI5*. These results are consistent with the notion that *ABI5* positively regulates ABA response genes, and, like *AtABI5*, *GhABI5* has conserved functions in seed dormancy.

### Silencing *GhABI5* by VIGS Reduced *Gladiolus* Corm Dormancy

**FIGURE 7:**
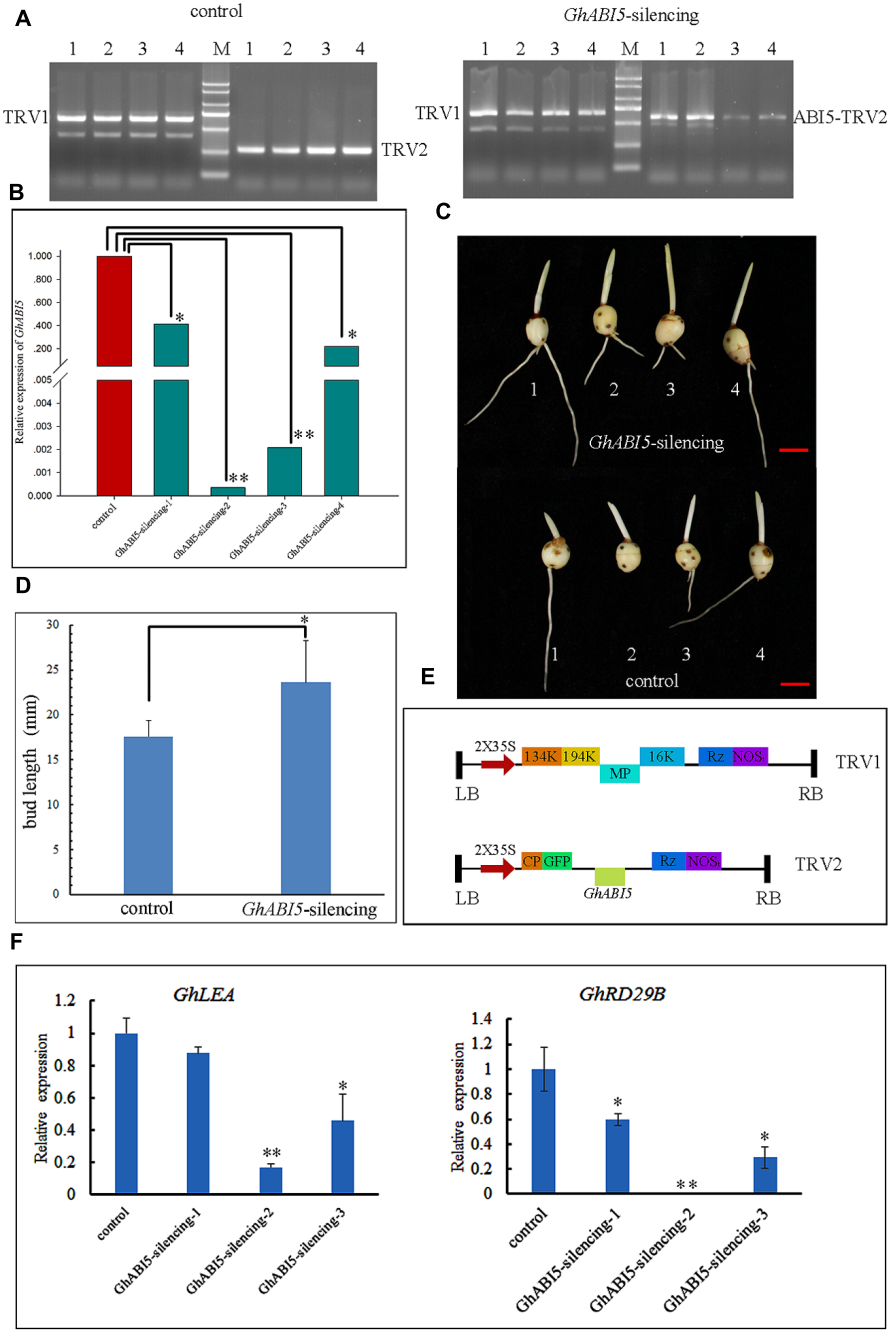
**Silencing of *GhABI5* promotes the germination of dormant cormels. (A)** Detection of *TRV1* (628 bp), *TRV2* (263 bp) and *ABI5-TRV2* (607 bp) by RT-PCR in silenced cormels. Left (the control), dormant cormels inoculated with *Agrobacterium* (*TRV1* and *TRV2*); Right, dormant cormels inoculated with *Agrobacterium* (*TRV1* and *ABI5-TRV2*). **(B)** Quantitative real-time PCR analysis of the expression level of *GhABI5* in the control and silenced lines. **(C)** Phenotype of silenced lines and controls. **(D)** Bud length of the control and silenced cormels (*n* = 8). **(E)** Schematic diagram of the *TRV1* and *TRV2–GFP* vectors. **(F)** The expression of *GhLEA* and *GhRD29B* in silencing cormels by Quantitative real-time PCR. (^∗^*P* < 0.05; ^∗∗^*P* < 0.01).

To further investigate the role of *GhABI5* in corm dormancy, *GhABI5* silencing was carried out through VIGS. In order to specifically silence *GhABI5*, we chose specific 5′ fragments (344 bp) to construct a tobacco rattle virus (TRV)-*GhABI5* vector (**Figure 7E**). Dormant cormels were infiltrated with *Agrobacterium* harboring *TRV-GhABI5*. After 10 days of cultivation, *TRV1* and *TRV2* were detected in the infiltrated cormels by RT-PCR (**Figure 7A**). We successfully detected the expression of *TRV1* and *TRV2* in plantlets. Then, we analyzed *GhABI5* gene expression in infected plantlets. Compared with the control, silenced plantlets showed low *GhABI5* expression, which was approximately 41% (silencing-1), 0.03% (silencing-2), 0.2% (silencing-3), and 22% (silencing-4) of the control’s expression (**Figure 7B**). Bud length was measured in the control and *GhABI5*-silenced plantlets. Silenced cormels had longer buds (23.62 ± 4.63 mm), approximately 1.34-fold longer than the buds of control cormels (17.61 ± 1.78 mm) (**Figures 7C,D**). The results indicate that *GhABI5* plays an important role in corm dormancy and inhibits cormel germination.

## Discussion

*Gladiolus* is one of the most commonly sold cut flowers around the world as well as in China, where *Gladiolus* sales rank second among bulb flowers (Wu et al., 2014). *Gladiolus* corms must go through a long period of growth cessation known as endo-dormancy, which is one of the main factors limiting its production value. In addition, endo-dormant corms require refrigeration storage, leading to high costs associated with electricity and labor. Therefore, elucidating the mechanism of corm dormancy will provide a theoretical basis for the production of weakly dormant *Gladiolus* cultivars through molecular breeding.

Endogenous hormones and other factors are responsible for inhibiting germination of the *Gladiolus* bud. However, this limitation can be overcome by cold storage, which reduces endogenous ABA content (**Figure 1A**) and results in earlier germination (**Figure 1D**). Exogenous ABA treatment maintains corm dormancy and inhibits bud germination, while fluridone promotes sprouting (**Figure 2**). Fluridone is a carotenoid biosynthesis inhibitor known to prevent ABA biosynthesis. However, it is not an ABA-specific inhibitor; our results show that fluridone inhibits root elongation, which has also been demonstrated in previous research (Hooker and Thorpe, 1998; Spollen et al., 2000). Although GA has proven to be important in seed dormancy, it may play a limited role in corm dormancy (Ram et al., 2002; Finkelstein et al., 2008). GA content increases only in the late dormancy-releasing period and fluctuates irregularly during dormancy breaking (**Figure 1B**). Previous studies have shown that GA has a limited effect on *Gladiolus* corm dormancy (Ram et al., 2002). In addition, corm dormancy and seed dormancy share a common physiological characteristic: they are both heavily regulated by ABA (Ahrazem et al., 2012; Finkelstein, 2013). This finding is also consistent with the transcriptome data of bud dormancy in perennials such as peach, leafy spurge and oak; in these plants, bud dormancy is also ABA-regulated (Horvath et al., 2008; Leida et al., 2012; Ueno et al., 2013). In the current study, VIGS was employed to knock down *GhABI5* expression. Although a target-specific fragment was chosen for siRNA-mediated *GhABI5* knockdown, it is also possible that other bZIP family members in the cormel were indirectly targeted. Therefore, the early sprouting phenotype and down-regulation of downstream gene expression (*GhLEA* and *GhRD29B*) in the silenced dormant cormels may be caused by down-regulation of multiple genes, including *GhABI5* (**Figure 7F**). These results suggest that ABA signaling is also involved in the regulation of corm dormancy (a vegetative organ). -= In *Arabidopsis*, *ABI5* is exclusively expressed in the seed (a reproductive organ). More specifically, it functions as an enhancer of seed dormancy as well as a repressor of seedling germination (Lopez-Molina et al., 2001; Garcia et al., 2008). Here, we have found that the expression of its homologous gene in *Gladiolus* (*GhABI5)* extends into dormant vegetative organs (dormant cormels, corms. and stolons), where it functions to enhance organ dormancy. Furthermore, *GhABI5* is able to complement the ABA insensitivity of *abi5-3* in *Arabidopsis*, can extend dormancy when overexpressed in transgenic lines (**Figure 6**) and induces the expression of downstream ABA response genes (*EM1*, *EM6*, and *RD29B*) (**Figure 6C**). It is clear that *GhABI5* has characteristics in common with *AtABI5*, including the induction of an ABA response and dormancy maintenance.

*Gh*ABI5 belongs to the bZIP superfamily of transcription factors, since it possesses a typical basic leucine zipper that binds DNA. Sequence analysis revealed that, among all members of the bZIP superfamily, *Gh*ABI5 shows the highest sequence similarity to *At*ABI5 (**Figure 3A**). Similar to the ABI5s in other species, *Gh*ABI5 also has the conserved C1, C2, and C3 domains (Zou et al., 2008; Zhou et al., 2013). The *Gh*ABI5 protein is localized in the nucleus and functions as a transcription factor, which is consistent with other homologous ABI5 proteins (Lopez-Molina et al., 2003; Zou et al., 2008; Zhou et al., 2013).

Abscisic acid is able to induce the expression of *GhABI5*, suggesting that this phytohormone may have an important physiological function in corm development. In addition, both drought treatment (**Figure 4D**) and corm desiccation (ABA increases during this period, **Figure 1A**) could increase the expression of *GhABI5*. Exogenous ABA also significantly enhances the transcription of *GhABI5*. Previous research has shown that ABA not only increases the expression level of *AtABI5* in *Arabidopsis* seeds, but also stabilizes the *At*ABI5 protein by relieving SnRK2 repression, which then phosphorylates *At*ABI5 (Brocard et al., 2002; Feng et al., 2014) and inhibits the E3 ligase KEG (Stone et al., 2006). In contrast, the ABA inhibitor fluridone could reduce the endogenous ABA content in dormant cormels, leading to down-regulation of *ABI5* expression (**Figure 4E**) and earlier germination (**Figure 2**). A similar situation was apparent following cold treatment (**Figures 1A** and **4C**). Cold storage has proven to be effective in breaking *Gladiolus* corm dormancy (Ginzburg, 1973). We hypothesize that this might occur through the modulation of ABA levels and ABA-associated signaling pathways within the plant. Long-term cold treatment diminishes endogenous ABA content, resulting in lower expression of *GhABI5* as well as a weakened ABA response. However, we noticed that the fluridone-treated cormels had shorter roots compared with the control. This finding is similar to that obtained from *Z. mays* L. seedlings, in which endogenous ABA levels correlated positively with increased rates of root elongation in most cultivars whose root tips contain a certain amount of ABA (Rivier and Pilet, 1981; Ng and Moore, 1985). We also found that *GhABI5* transcript levels are highly abundant in the stamen (reproductive organ). In rice, *OsABI5* is expressed in mature pollen and contributes to plant fertility (Zou et al., 2008). In *Brassica oleracea*, *BoABI5* is specifically expressed in the flower (Zhou et al., 2013).

The *GhABI5* promoter harbors numerous *cis*-acting regulatory elements, which mediate plant responses to developmental cues and to stress-related hormones. ABA up-regulates *GhABI5* expression, as evidenced by the transient expression of *GhABI5:GUS* in *Gladiolus* petals. Several conserved Ebox/ABRE (CANNTG) and DPBF (ACACNNG) motifs are likely responsible for the up-regulation of *GhABI5* expression upon ABA treatment (Stalberg et al., 1996; Kim et al., 1997). It is reported that MYB and MYC transcription factors contribute to ABA-related responses such as drought and water stress (Abe et al., 1997, 2003; Cominelli et al., 2005). Therefore, MYBs and MYCs may regulate *GhABI5* expression in an ABA-dependent manner during corm dormancy and drought stress. Of particular note, the RY/G box (CATGCA) motif occurs in the *GhABI5* promoter sequence (Ezcurra et al., 2000). Previous research has shown that ABI3 specifically binds to the RY/G box motif and up-regulates *AtABI5* expression, suggesting that other transcription factors involved in ABA signaling may regulate corm dormancy (Lopez-Molina et al., 2002). Moreover, our study suggests that cold treatment can reduce the expression of *GhABI5*. The LTRE motif, which is found in the promoter of cold-inducible genes, is present in the promoter of *GhABI5*; this suggests potential crosstalk between cold and ABA, mediated by *GhABI5* (Baker et al., 1994). Future work will focus on the identification of hormone-regulated transcription factors that modulate *GhABI5* expression and affect corm dormancy.

Taken together, our results provide novel insight into the molecular mechanisms underlying corm dormancy. This physiological process is regulated through ABA signal transduction, in which *Gh*ABI5 has a positive response to ABA and enhances corm dormancy. We suspect that similar cellular and molecular mechanisms exist among various types of dormant organs (seed, modified stem and modified root).

## Author Contributions

JW and MY conceived and designed the research. MY and JH conducted experiments. JW and SS performed the assays. JS, XL, and BG helped take the photographs. Chen L and CW analyzed data. CL and FZ helped take care of plants. JW, MY, JH, and EV wrote the manuscript. All authors read and approved the manuscript.

## Conflict of Interest Statement

The authors declare that the research was conducted in the absence of any commercial or financial relationships that could be construed as a potential conflict of interest.

## Footnotes

1 http://au.expasy.org/tools/

2 https://sogo.dna.affrc.go.jp/cgi-bin/sogo.cgi?lang=en&pj=640&action=page&page=newplace

3 http://www.arabidopsis.org
