# Supplementary material for: Gladiolus hybridus ABSCISIC ACID INSENSITIVE 5 (GhABI5) is an important transcription factor in ABA signaling that can enhance Gladiolus corm dormancy and Arabidopsis seed dormancy
Source: Front Plant Sci. 2015 Nov 3;6:960. doi: 10.3389/fpls.2015.00960 (PMC4630654; doi:10.3389/fpls.2015.00960)
Supplement: Supplementary file 1 [file Image_1.PDF]

Supplementary Material

Gladiolus hybridus ABSCISIC ACID INSENSITIVE 5 (*Gh*ABI5) is an important transcription factor in ABA signaling that can enhance Gladiolus corm dormancy and Arabidopsis seed dormancy.

Jian Wu[1*], Shanshan Seng[1*], Juanjuan Sui[1], Eliana Vonapartis[2,3], Xian Luo[4], Benhe Gong[1], Chen Liu[1], Chenyu Wu[1], Chao Liu[1], Fengqin Zhang[1], Junna He[1†], Mingfang Yi[1†]

[1] Beijing Key Laboratory of Development and Quality Control of Ornamental Crops, Department of Ornamental Horticulture and Landscape Architecture, China Agricultural University, Beijing, China
[2] Department of Biological Sciences, University of Toronto, Toronto, Ontario, Canada
[3] Department of Cell and Systems Biology, University of Toronto, Toronto, Ontario, Canada
[4] College of Horticulture, Sichuan Agricultural University, Yucheng District, Ya'an, Sichuan, China

[*]These authors contributed equally to this work.

[†] **Correspondence:** Junna He and Mingfang Yi, Beijing Key Laboratory of Development and Quality Control of Ornamental Crops, Department of Ornamental Horticulture and Landscape Architecture, China Agricultural University, Beijing 100193, China

E-mail: hejunna@cau.edu.cn; ymfang@cau.edu.cn

Tel.: +86 10 62733817; fax: +86 10 62733603

Supplementary Figures

```
-1470                                                                           CCTT
        RY motif MYC                                             GATA box
-1400 GCGCCCATGGCATGCATTTGGTACCGTCTCCACAGCTAATGAAGTTTGTTGGATATGGGGCCAAAGCAAG
       GATA box
-1330 CCAGGATAATGATGTCAGAATTAGTGGGCCCCTTCAGCCTAGGGAGAGAAAGAAGAGAGAGATGAGAGAG
        GATA box                                       GATA box W box
-1260 CTGCCATGTATATAGATATAGAAAGAGAGAGAGAGGAGAGAGAGAGATATGACAGAGCAGCAGCTCACTT
        GATA box   GATA box  ARR                                  TAAAG motif
-1190 AAAGAGAGAGGAGACAGCGCTTTGATAGGTCGATTCTCCTTTCTCCTTAGTCTCTCTAAAGAAGTCTCAT
        TATA box MYC          MYC                        GATA box        TATA box
-1120 ATTTATTTCATTTGGTTAGAATTGAGAGCATTTGCTTGTCTAATTTGAGATAGTAATCTGAATTTTATTT
        CAAT box TAAAG motif
-1050 TATAAGTGCAATATAAAGTTAGAATTTGAAGGTTTCTTCTTCTCTTACCTACTTTCTTTGTCTGCTTTTT
                                GATA box        GATA box
-980  GTTCATCAAACTGCACTTATTATGATGTGATAATCGAGATCTTGATAGATCATCACCTCTGTCATTTATT
                  CAAT box ARR        MYC       ARR
-910  GTTCACATCCTTATCTCAATCTTTGATTCTACTTCATTTGAGGATGATTGTATGAAATTACTGCATAATT
                                                  Pryimiding box
-840  TACTTTTCTGTCATAATGTTGTAATTTTTTTTTCTTTTTCTTTCCTTTTAGCATGGAAATTAAGAGTAGG
                                TATA box
-770  GAAGTAGAAGTATTTAACTTGTGGTTTTTATTTGTTAGCTGAGAAAGGAAAACTAGTTGTAGCCGCTCAC
             ARR          ARR
-700  AGTGGCATTCGATTTCTTGTCCCTGATTTGAAATATCAGTGGCTGAGTGGGGTGTTATTCTGGACTGATC
                              W box                   CAAT box              Inr
-630  TATTCGCGTAATGAATGGCCAACATGACCTATCTTATATATCTATTGCAATTCTCTTTTGCATTCTTCAT
       GATA box           TATA box MYB                                  MYC
-560  TTTTGGATATTTCTACATTCATATAAATTAACTGGTTATGTAATTTTCCAGTCGTCACCTTTCCATTTGA
               MYC         CAAT box           ERF
-490  GTGGTGTTTTCATGTGTCTTTTCAATTTTTTGAAAAATTCAAAATAAAATAATACATGTTCAGTGTCGCT
                                MYC              MYC        Inr
-420  CTGGCTAATGCATTAATCTTCACCTTTCCATTTGAGTGGTGCTTTCATGTGTCTTTTCATTTTATGAAAA
                               ARF
-350  GTTCAAAATAAATAATACATGTTTAGTGTCTCTCTGGCTAATCCATTAATCTTGTGTTAGTCCCTTGGTC
                                                               TATA box TATA box
-280  AGCACTTTTCAACAAAAAAAAAAAAAATAACAGTTTGGGAAAATTAAAAAGAAGACCTATATAAATTACTG
               ARR               MYB        CBF
-210  TTTTTCCTACTGATTTTGAATGTATTGTACTGTTGTAAGAAATCGACATGCTGAGTTATATTTTAACTCT
       ARR
-140  AGATTTTTCTACATGGCCGGTGTGCTTTTAACTCATTGCCTACCGTACCAGTGTGTAATAAATAGAAATA
                    MYB                                                     DPBF
-70   GAAGATCAGAAATCCAACGGCTGAAATTAAACGGTGCTGGCGTAAAATGCCAGACTTTGTCATCGTACAC
                               GATA box TATCCAY motif  Inr            MYC
+1    ATGTCTTCAAGATCTTATCTGCATGGATATCCACTGTCCTCACTCCTAACCTCCGCATTTGTCGCCATCT
         LTRE       MYB        MYB  MYB
+71   AAATCCGACGGTCCACAGTTACCAAACCTAACCATCACACGGTCCACACCACACGCATCTACTGTACTGT
                              TAAAG motif    MYC  MYB
+141  CACTGTGCTAGTCTTTCGTATTAAAGACCGCCTTCACCTGTTATGCTTTCTGCCCATTCTTACTTTTGTC
                Pryimiding box                       ARR
+211  AAAAAAATTCTCCTTTTTTTTTGCAGTGATCAAGAAAATTTGGATTGGAAAAGAAAGAAGAGGTTTAGCC
+281  GGCAATG
```

Fig. S1: Sequence and putative cis-elements within the *GhABI5* promoter.

Numbers indicate the positions relative to the transcription start site (+1). The translation start site is bolded, and the ATG of its ORF is boxed. Some important putative cis-elements are marked with a gray color and labeled.

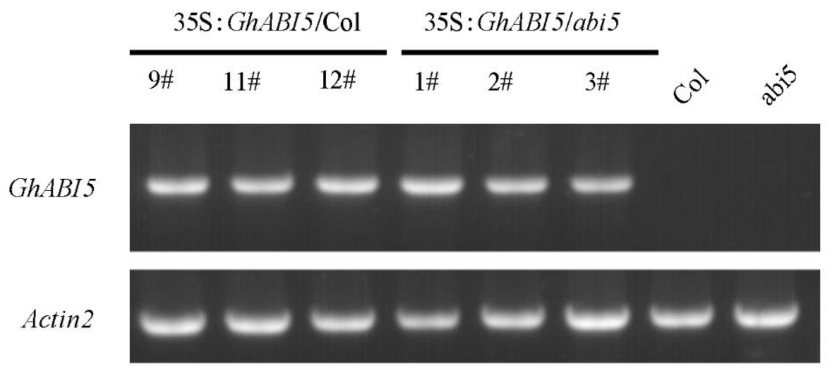

Fig. S2: Overexpression of *GhABI5* in Arabidopsis.

Analysis of *GhABI5* transcripts by RT-PCR in the Col, *abi5* and transgenic plants, respectively. *Actin2* was used as an internal control.

Table S1 Primer sequences used in this study.

Primer	Forward sequence（5′-3′）	Reverse sequence（5′-3′）
**For isolation of *GhABI5***		
GhABI5-DP	GGBTCCATGAACATGGACGAG	KCCAVACCTCVTCVACVGTCTT
GhABI5-3’	ACATCTGGAACGTCGAGGAAGCG	GCTGTCAACGATACGCTACGTAACG
	ATCAGCAGCAGACGTCACTACAAC	CGCTACGTAACGGCATGACAGTG
GhABI5-5’		CCGGAGATCCCAGCCTCAGCTGGTACGA
GhABI5-CDS	GAAGAGGTTTAGCCGGCAATGG	CTAGCTATGGTGGGTGCAGATTTAC
GhABI5-1300	GCTCTAGAATGGCGTCCCCGGCGGAGAG	GGGGTACCCCATGGTGCACTGTTTGCTCGAC
**For isolation of *GhABI5* upstream regulatory sequence**		
SP1		GTTCTGTATCTCGTCCAGCGTCAG
SP2		CTCGCTCTCATCCACCTTACTCTCCGC
SP3		TTGACAAAAGTAAGAATGGGCCCCCATG
**For vector construct of *GhABI5* promoter in GUS staining**		
GhABI5-Pro	CCCAAGCTTCCTTGCGCCCATGGCATGCATTTGG	CGCGGATCCTGCCGGCTAAACCTCTTCTTTCTTTTCC
**For qRT-PCR analysis in *Gladiolus hybridus* and Arabidopsis**		
GhABI5	GGACAAAGGCAACGAGGAAAC	TCGGGCAGTCTTCTCCACAT
GhLEA	TCCGTTATCGCCAAGTCCAA	CGGATCCATCTCGACAACCC
GhRD29B	GGGACGTCAGTGAAGGAGTT	TCTGATCCGTTGTCTCTTGC
GhACTIN	ACTGCAGAGCGGGAAATTGT	CCAATCAGGGATGGCTGGAA
AtEM1	CGAGCTACTAGTGTCCGCTGCA	GTAAAACCAACCGGCAACCGCA
AtEM6	ATGGCGTCTCAACAAGAGAA	TTAGGTCTTGGTCCTGAATTTG
AtRD29B	AGAAGGAATGGTGGGGAAAG	CAACTCACTTCCACCGGAAT
AtACTIN2	GGTAACATTGTGCTCAGTGGTGG	AACGACCTTAATCTTCATGCTGC
**For *GhABI5* silencing**		
GhABI5-segment	GCTCTAGAATGGCGTCCCCGGCGGAGAG	GGGGTACCTGAAGTGACGTCTGCTGCTG
TRV1	TTACAGGTTATTTGGGCTAG	CCGGGTTCAATTCCTTATC

TRV2	TGGGAGATGATACGCTGTT	CCTAAAACTTCAGACACG

Table S2 ABI5 homologous genes used in the sequence analysis from NCBI database.

**Species**	**Name**	**Accession no.**
Arabidopsis thaliana	*AtABI5*	NM_129185.3
Brassica napus	*BnABI5*	KC414029.1
Elaeis guineensis	*EgABI5*	JN003476.1
Fragaria vesca	*FvABI5*	XM_004305297.1
Glycine max	*GmABI5*	XM_003553547.2
Hordeum vulgare	*HvABI5*	AY150676.1
Morus notabilis	*MnABI5*	XP_010101577.1
Oryza brachyantha	*OsABI5*	XM_006656698.1
Prunus mume	*PmABI5*	XP_008241247.1
Setaria italica	*SiABI5*	XM_004964845.1
Triticum aestivum	*WABI5*	AB362820.1
Zea mays	*ZaABI5*	NM_001157477.1

Table S3 *Cis*-regulatory elements present in the *GhABI5* promoter region

Cis-element	Sequence core	Number	Conditions	Reference
ASF1	TGACG	1(-)	auxin, SA, disease resistance	(Klinedinst et al., 2000;Redman et al., 2002;Despres et al., 2003)
ARF	TGTCTC	1(+), 1(-)	auxin	(Ulmasov et al., 1999)
CRT/DRE	RYCGAC	1 (+), 1(-)	drought	(Svensson et al., 2006)
DPBF	ACACNNG	1 (+), 5(-)	ABA	(Kim et al., 1997)
EBOX/ABRE	CANNTG	11(+), 11(-)	ABA	(Stalberg et al., 1996)
ERE	AWTTCAAA	1(+), 2(-)	ethylene	(Tapia et al., 2005)
GARE	TAACAAR	1(-)	GA	(Ogawa et al., 2004)
LTRE	CCGAC	1(+)	ABA, drought, cold	(Baker et al., 1994)
MYB1	WAACCA	1(+), 3(-)	ABA	(Abe et al., 2003)
MYB2	YAACKG	2(+), 1(-)	water stress, ABA	(Urao et al., 1993)
MYB	CTAACCA	3(+), 3(-)	drought, ABA, water stress	(Abe et al., 1997)
MYC	CATGTG	2(+), 1 (-)	water stress	(Simpson et al., 2003)
PYRIMIDINE	CCTTTT	1(+), 1 (+)	GA, sugar	(Morita et al., 1998;Mena et al., 2002)
RY/G box	CATGCA	1(+), 1(-)	ABA	(Ezcurra et al., 2000)
TATCCA	TATCCA	2(-), 1(+)	GA, sugar	(Chen et al., 2006)
WBOX	TTGAC	1(-)	SA	(Yu et al., 2001)
WRKY710S	TGAC	2(+), 8(-)	GA	(Yu et al., 2001)

Supplementary Referencee

Abe, H., Urao, T., Ito, T., Seki, M., Shinozaki, K., and Yamaguchi-Shinozaki, K. (2003). Arabidopsis AtMYC2 (bHLH) and AtMYB2 (MYB) function as transcriptional activators in abscisic acid signaling. *Plant Cell* 15**,** 63-78. doi: Doi 10.1105/Tpc.006130.

Abe, H., Yamaguchi-Shinozaki, K., Urao, T., Iwasaki, T., Hosokawa, D., and Shinozaki, K. (1997). Role of arabidopsis MYC and MYB homologs in drought- and abscisic acid-regulated gene expression. *Plant Cell* 9**,** 1859-1868. doi: 10.1105/tpc.9.10.1859.

Baker, S.S., Wilhelm, K.S., and Thomashow, M.F. (1994). The 5'-Region of Arabidopsis-Thaliana Cor15a Has Cis-Acting Elements That Confer Cold-Regulated, Drought-Regulated and Aba-Regulated Gene-Expression. *Plant Mol Biol* 24**,** 701-713. doi: Doi 10.1007/Bf00029852.

Chen, P.W., Chiang, C.M., Tseng, T.H., and Yu, S.M. (2006). Interaction between rice MYBGA and the gibberellin response element controls tissue-specific sugar sensitivity of alpha-amylase genes. *Plant Cell* 18**,** 2326-2340. doi: DOI 10.1105/tpc.105.038844.

Despres, C., Chubak, C., Rochon, A., Clark, R., Bethune, T., Desveaux, D., et al. (2003). The Arabidopsis NPR1 disease resistance protein is a novel cofactor that confers redox regulation of DNA binding activity to the basic domain/leucine zipper transcription factor TGA1. *Plant Cell* 15**,** 2181-2191. doi: Doi 10.1105/Tpc.012849.

Ezcurra, I., Wycliffe, P., Nehlin, L., Ellerstrom, M., and Rask, L. (2000). Transactivation of the Brassica napus napin promoter by ABI3 requires interaction of the conserved B2 and B3 domains of ABI3 with different cis-elements: B2 mediates activation through an ABRE, whereas B3 interacts with an RY/G-box. *Plant J* 24**,** 57-66. doi: DOI 10.1046/j.1365-313x.2000.00857.x.

Kim, S.Y., Chung, H.J., and Thomas, T.L. (1997). Isolation of a novel class of bZIP transcription factors that interact with ABA-responsive and embryo-specification elements in the Dc3 promoter using a modified yeast one-hybrid system. *Plant J* 11**,** 1237-1251. doi: DOI 10.1046/j.1365-313X.1997.11061237.x.

Klinedinst, S., Pascuzzi, P., Redman, J., Desai, M., and Arias, J. (2000). A xenobiotic-stress-activated transcription factor and its cognate target genes are preferentially expressed in root tip meristems. *Plant Mol Biol* 42**,** 679-688. doi: Doi 10.1023/A:1006332708388.

Mena, M., Cejudo, F.J., Isabel-Lamoneda, I., and Carbonero, P. (2002). A role for the DOF transcription factor BPBF in the regulation of gibberellin-responsive genes in barley aleurone. *Plant Physiol* 130**,** 111-119. doi: 10.1104/pp.005561.

Morita, A., Umemura, T., Kuroyanagi, M., Futsuhara, Y., Perata, P., and Yamaguchi, J. (1998). Functional dissection of a sugar-repressed alpha-amylase gene (RAmy1 A) promoter in rice embryos. *FEBS letters* 423**,** 81-85.

Ogawa, M., Hanada, A., Yamauchi, Y., Kuwahara, A., Kamiya, Y., and Yamaguchi, S. (2004). Gibberellin biosynthesis and response during Arabidopsis seed germination (vol 15, pg 1591, 2003). *Plant Cell* 16**,** 783-783.

Redman, J., Whitcraft, J., Johnson, C., and Arias, J. (2002). Abiotic and biotic stress differentially stimulate as-1 element activity in Arabidopsis. *Plant Cell Rep* 21**,** 180-185. doi: DOI 10.1007/s00299-002-0472-x.

Simpson, S.D., Nakashima, K., Narusaka, Y., Seki, M., Shinozaki, K., and Yamaguchi-Shinozaki, K. (2003). Two different novel cis-acting elements of erd1, a clpA homologous Arabidopsis gene function in induction by dehydration stress and dark-induced senescence. *Plant J* 33**,** 259-270.

Stalberg, K., Ellerstom, M., Ezcurra, I., Ablov, S., and Rask, L. (1996). Disruption of an overlapping E-box/ABRE motif abolished high transcription of the napA storage-protein promoter in transgenic Brassica napus seeds. *Planta* 199**,** 515-519.

Svensson, J.T., Crosatti, C., Campoli, C., Bassi, R., Stanca, A.M., Close, T.J., et al. (2006). Transcriptome analysis of cold acclimation in barley Albina and Xantha mutants. *Plant Physiol* 141**,** 257-270. doi: DOI 10.1104/pp.105.072645.

Tapia, G., Verdugo, I., Yanez, M., Ahumada, I., Theoduloz, C., Cordero, C., et al. (2005). Involvement of ethylene in stress-induced expression of the TLC1.1 retrotransposon from Lycopersicon chilense Dun. *Plant Physiol* 138**,** 2075-2086. doi: DOI 10.1104/pp.105.059766.

Ulmasov, T., Hagen, G., and Guilfoyle, T.J. (1999). Dimerization and DNA binding of auxin response factors. *Plant J* 19**,** 309-319. doi: DOI 10.1046/j.1365-313X.1999.00538.x.

Urao, T., Yamaguchishinozaki, K., Urao, S., and Shinozaki, K. (1993). An Arabidopsis Myb Homolog Is Induced by Dehydration Stress and Its Gene-Product Binds to the Conserved Myb Recognition Sequence. *Plant Cell* 5**,** 1529-1539. doi: DOI 10.1105/tpc.5.11.1529.

Yu, D., Chen, C., and Chen, Z. (2001). Evidence for an important role of WRKY DNA binding proteins in the regulation of NPR1 gene expression. *Plant Cell* 13**,** 1527-1540.
